# B cells expressing the IgA receptor FcRL4 participate in the autoimmune response in patients with rheumatoid arthritis

**DOI:** 10.1016/j.jaut.2017.03.004

**Published:** 2017-07

**Authors:** Khaled Amara, Elizabeth Clay, Lorraine Yeo, Daniel Ramsköld, Julia Spengler, Natalie Sippl, James A. Cameron, Lena Israelsson, Philip J. Titcombe, Caroline Grönwall, Ilfita Sahbudin, Andrew Filer, Karim Raza, Vivianne Malmström, Dagmar Scheel-Toellner

**Affiliations:** a Rheumatology Unit, Department of Medicine, Karolinska University Hospital, Karolinska Institutet, SE-17176 Solna, Stockholm, Sweden; b Rheumatology Research Group, RACE AR UK Centre of Excellence in RA Pathogenesis, Institute of Inflammation and Ageing, College of Medical and Dental Sciences, University of Birmingham, Birmingham B15 2TT, UK; c Department of Rheumatology, Sandwell and West Birmingham Hospitals NHS Trust, Birmingham UK

**Keywords:** Antibodies, Autoimmunity, B cells, Rheumatoid arthritis, FcRL4, IRTA1

## Abstract

The clinical efficacy of B cell targeting therapies highlights the pathogenic potential of B cells in inflammatory diseases. Expression of Fc Receptor like 4 (FcRL4) identifies a memory B cell subset, which is enriched in the joints of patients with rheumatoid arthritis (RA) and in mucosa-associated lymphoid tissue. The high level of RANKL production by this B cell subset indicates a unique pathogenic role. In addition, recent work has identified a role for FcRL4 as an IgA receptor, suggesting a potential function in mucosal immunity. Here, the contribution of FcRL4+ B cells to the specific autoimmune response in the joints of patients with RA was investigated.

Single FcRL4+ and FcRL4- B cells were sorted from synovial fluid and tissue from RA patients and their immunoglobulin genes characterized. Levels of hypermutation in the variable regions in both populations were largely consistent with memory B cells selected by an antigen- and T cell-dependent process. Recombinant antibodies were generated based on the IgH and IgL variable region sequences and investigated for antigen specificity. A significantly larger proportion of the recombinant antibodies generated from individual synovial FcRL4+ B cells showed reactivity towards citrullinated autoantigens. Furthermore, both in analyses based on heavy chain sequences and flow cytometric detection, FcRL4+ B cells have significantly increased usage of the IgA isotype. Their low level of expression of immunoglobulin and plasma cell differentiation genes does not suggest current antibody secretion. We conclude that these activated B cells are a component of the local autoimmune response, and through their RANKL expression, can contribute to joint destruction. Furthermore, their expression of FcRL4 and their enrichment in the IgA isotype points towards a potential role for these cells in the link between mucosal and joint inflammation.

## Highlights

• Memory B cells expressing the IgA receptor FcRL4 are found in the joints of patients with RA.
• B cell receptors expressed on synovial FcRL4+ B cells more frequently belong to the IgA class.
• Among recombinant antibodies cloned from FcRL4+ B cells there is more reactivity with citrullinated proteins.
• The gene transcription profile of FcRL4+ B cells shows a low level of differentiation to plasma cells.
• These cells may be involved in the link between mucosal and joint autoimmunity.

## 1 Introduction

Rheumatoid Arthritis (RA) is a chronic autoimmune inflammatory disease that affects ca. 1% of the human population. The anti-inflammatory effect of depletion of B cells with the CD20-targeting antibody rituximab confirms a role of these cells in mediating RA disease activity [1]. 60–80% of RA patients have B cell responses characterized by the production of autoantibodies (ACPAs) recognizing several citrullinated proteins, mainly fibrinogen, vimentin and α-enolase [2]. These humoral autoimmune responses are primarily composed of IgG but can also contain other isotypes including IgA [3]. In a recent study, we found that immunoglobulins expressed by B cells isolated from the RA joint exhibit a bias towards recognition of citrullinated autoantigens [4].

A growing body of data suggests that ACPAs can contribute to disease by stimulating osteoclast differentiation, complement activation and immune complex formation [5], [6], [7], [8]. However, whilst B cell depletion by the anti-CD20 antibody rituximab reduces RA disease activity and erosive damage [1], [9], the B cell immuno-modulatory effects of rituximab do not necessarily coincide with changes in autoantibody levels [10]. Therefore, the whole range of contributions of B cells to disease pathology remains undefined. Multiple mechanisms are plausible, including autoantibody production, antigen presentation and cytokine secretion [11], [12].

We recently identified a subset of memory B cells in the RA synovium, which expresses high levels of RANKL, a cytokine with key roles both in bone destruction and development of lymphoid structures [13], [14], [15]. This cytokine-producing B cell subset is defined by expression of Fc receptor-like 4 (FcRL4) on their surface. As synovial RANKL expression is significantly reduced under B cell targeting therapy [16], removal of FcRL4+ B cells may explain some of the autoantibody-independent effects of B cell depletion.

FcRL4 is a transmembrane protein with a high level of homology to Fc receptors. In particular, it is closely related to FcγRIIB [17], [18]. While the other members of the *FcRL* gene family are evolutionary conserved, *FcRL4* is restricted to higher primates. Initially, FcRL4 was thought to have an entirely inhibitory function on B cell receptor signaling. However, more recent data point towards an additional role in sensitizing B cells to TLR9 mediated NFkB activation, suggesting that the consequences of FcRL4 ligation are context dependent [19], [20], [21].

B cells expressing FcRL4 were first described as a distinct memory B cell subset in human tonsils [22], [23]. These cells accumulate in the epithelium of mucosa associated lymphoid tissue (MALT) and are less frequently found in the B cell rich regions of follicles and germinal centers [22], [24]. Although FcRL4+ B cells display an activated, highly proliferative phenotype [23], the antigens they recognize in the mucosa have not yet been identified. There is little understanding of their contribution to mucosal inflammation beyond the observation that FcRL4 can act as a low affinity receptor for IgA [25]. Given that FcRL4+ B cells are also enriched in the RA joint and produce cytokines that could contribute to joint destruction [13], [14], we hypothesized that these cells may recognize local citrullinated autoantigens. Here, we investigated the immunoglobulin (Ig) isotype and the characteristics of the Ig variable region genes expressed in FcRL4+ B cells isolated from RA synovial fluid and tissue. Recombinant monoclonal antibodies were generated from single-cell isolated transcripts, to determine whether the surface Ig of FcRL4+ B cells can recognize citrullinated autoantigens. Furthermore, we explored the functional role of FcRL4+ B cells by comparing their transcriptional profile to FcRL4- B cells sorted from the same joints.

## 2 Material and methods

### 2.1 Patients

A total number of 19 synovial fluid (SF) and 2 synovial tissue (ST) samples were included in this study. Samples were obtained from patients fulfilling 1987 American College of Rheumatology (ACR) criteria for RA [26]. ST samples were obtained at the time of joint-replacement surgery. A summary of patient characteristics is shown in Table 1. A more detailed set of characteristics including current and recent immunosuppressive therapy is shown in the supplementary table 1 [27]. The cell numbers yielded from individual samples was too low to perform all experiments with material from the same patients. The samples used for the individual experiments are identified in supplementary table 1 [27]. The study was conducted in compliance with the Helsinki declaration, ethical approval was obtained from the local ethics committee and all subjects gave informed, written consent.

**Table 1:** Clinical characteristics of RA patients who provided synovial fluid or synovial tissue. RF, rheumatoid factor; CCP, cyclic citrullinated peptide; CRP, C reactive protein; ESR, erythrocyte sedimentation rate, DAS28, disease activity score 28. More detailed clinical characteristics can be found online in the supplementary data paper [27].

	Synovial fluid samples	Synovial tissue samples
Number	19	2
Disease duration (yrs); median (IQR)	3 (1.5–10)	28.5
Female; n (%)	14 (74)	1
Age yrs; median (IQR)	60 (47.5–65.5)	69.5
CRP (mg/l); median (IQR)	20 (13–57)	5.5
ESR (mm/h); median (IQR)	30 (15–55)	21
DAS28 CRP; median (IQR)	5.4 (4.6–6.2)	3.5
DAS28 ESR; median (IQR)	5.9 (5.1–6.7)	4.7
Swollen joint count of 28; median (IQR)	6 (2–10)	2
Tender joint count of 28; median (IQR)	7.5 (5–14.7)	7

### 2.2 Flow cytometry and single B cell sorting

Synovial fluid was incubated with 1000 U ml^−1^ hyaluronidase (Hyalase^TM^, Wockhardt UK Ltd) at 37 °C for 15 min to reduce viscosity. Mononuclear cells were isolated from SF using density gradient centrifugation and from mechanically dissociated ST as previously described [28]. Cells from 4 SF and 2 ST samples (Suppl table 1; patients 1–6) [27] were stained for flow cytometry with mouse monoclonal antibodies against CD19 (Biolegend 302234) and FcRL4 (Biolegend 340204). A total of 96 single B cells from CD19+FcRL4+ and CD19+FcRL4- populations were sorted from each SF and ST samples using a MoFlo cell sorter (Dako) into 96-well PCR plates as previously described [29], [30]. Purity of sorted cells was in excess of 95%.

For detection of IgA B cell receptors, mononuclear cells were isolated from SF of 11 further RA patients (Suppl. table 1; patients 7–17) [27]. Surface receptor-bound antibodies were first removed by incubating SF B cells in 0.1 M glycine buffer at pH3 for 90 s. Cells were washed, labeled with antibodies to IgA (Miltenyi), FcRL4 (Biolegend) and CD19 (Biolegend) and analyzed on a Cyan ADP flow cytometer (Beckman).

### 2.3 PCR amplification and expression vector cloning

cDNA from the single sorted FcRL4+ and FcRL4- B cells was synthesized in a total volume of 15 μl in the original 96-well PCR plate using the SuperScript III RT (Gibco Invitrogen). Individual IgH (γ, α or μ) and IgL chain (κ or λ) gene rearrangements were amplified independently, using the cDNA as template, by two successive rounds of PCR (50 cycles each) using primers as previously described [29], [30]. For identification of Ig isotypes, amplification of IgH chains with reverse primers specific for the constant regions of all human Ig classes and sequencing was used. Cloning of the Ig genes into expression vectors was performed as described before [29], [30]. Briefly, digested PCR products were cloned in frame into expression vectors containing human Igγ1, Igκ1, or Igλ1 constant regions. Ligation reactions were performed using the quick ligase kit (New England Biolabs, Inc.). Expression vectors were transformed into DH5α bacteria (Gibco Invitrogen) and sequentially isolated using QIAprep Spin Miniprep kit (Qiagen). All PCR products were sequenced after cloning to confirm identity with the original PCR product and to ensure that clones with mutations introduced by the error-prone Taq polymerase were excluded from the analyses.

### 2.4 Ig gene sequence analysis

Matching IgH and IgL amplicons obtained from individual cells were sequenced (Eurofins MWG Operon) and analyzed for Ig gene usage, the complementarity-determining region 3 (CDR3) features, and number of V gene somatic hypermutations (SHMs) by IgBLAST comparison (http://www.ncbi.nlm.nih.gov/igblast/) and ImMunoGeneTics (IMGT) information system, (http://imgt.cines.fr). The IgH CDR3 length was determined as indicated previously [31]. Percent V gene mutation was calculated based on the number of V gene nucleotide mismatches determined by IgBLAST/IMGT, and the length of the V gene that was sequenced. The number of positively and negatively charged amino acids was determined for each IgH CDR3 region. Numbers of V gene SHMs were counted for framework regions (FWR1, FWR2, and FWR3) and CDRs (CDR1 and CDR2) inclusively. Replacement (R) and silent (S) mutation frequencies in FWRs and CDRs were calculated for each region based on the absolute number of V gene nucleotides in all analyzed sequences as defined by IMGT. IgG isotype subclasses were determined using the IMGT database. Immunoglobulin Analysis Tool (IgAT) was used to calculate the probability of antigen-driven selection within the Ig repertoire of the monoclonal antibodies as previously described [32].

### 2.5 Antibody production and purification

Monoclonal antibodies were produced by transient transfection in high-density suspension cultures of Expi293F™ cells using polyethylenimine (PEI)-Max (Polysciences) as transfection agent in a total of 38 μg of vector DNA (19 μg of IgH and corresponding IgL, each) as recommended by the manufacturer (Gibco Invitrogen). Monoclonal antibodies were purified on Protein G Sepharose 4 Fast Flow (GE Healthcare) and eluted with 0.1 M glycine buffer, pH 3.0, into neutralization buffer (1 M Tris-HCl, pH 8.0). Monoclonal antibody concentrations were determined by anti–human IgG1 ELISA using human monoclonal IgG1 as standard (Sigma-Aldrich) as previously described [29], [30].

### 2.6 Antibody specificities

#### 2.6.1 Assessment of IgG antibody reactivity against citrullinated peptides

The Anti–α-enolase reactivity was determined by ELISA as described, briefly: 96-well Nunc plates were coated with 2.5 μg ml^−1^ of the α-enolase peptide 1 in its native (REP-1) or citrullinated (CEP-1) form [33], [34]. Anti-vimentin and -fibrinogen reactivity was determined by ELISA as described previously [35]. In brief, streptavidin-coated high binding–capacity 96-well plates (Thermo Fisher Scientific) were coated with 1 μg ml^−1^ of biotinylated vimentin (amino acids 60–75) or fibrinogen (amino acids 36–52) peptides in their native and citrullinated forms. In all ELISAs, purified monoclonal antibodies were diluted in blocking buffer and used at a concentration of 5 μg ml^−1^. HRP-conjugated goat anti–human IgG (Jackson ImmunoResearch Laboratories, Inc.) was used as the detecting antibody and visualized using the chromogenic substrate 3,3′,5,5′-tetramethylbenzidine (Bio-Rad Laboratories). Reactivity was detected at 450 nm with a reference of 650 nm. A standard curve constructed with a serum sample positive for specific antigen was included on each plate and was further used to translate OD values to AU values, where the control maximum corresponds to 1000 AU. The AU values were divided by antigen-specific detection cutoffs based on prior experience with a larger set of clones. Reactivity with the synthetic cyclic citrullinated peptides (CCP2) was determined with the commercially available ELISA kit (Eurodiagnostica).

### 2.7 Gene expression analysis

RNA sequencing was carried out on sorted FcRL4+ and FcRL4- B cells from 4 additional SF samples (supplementary data paper table 1) [27]. Synovial fluid mononuclear cells were sorted into CD19+/FcRL4+ and CD19+/FcRL4- populations into PBS supplemented with 10% BSA/5 mM EDTA. RNA was isolated using the PicoPure RNA extraction kit (ThermoFisher). Preamplification prior to Illumina Truseq library preparation was performed using the SMARTer amplification using olig(dT) primed reactions (Clontech cat# 635001). Library prep was carried out using the Illumina TruSeq Stranded prep kit and sequenced on the Illumina HiSeq 2000/2500 platform (100 bp read length, paired end) using reagent kit v3.

We initially analyzed the data using the Tuxedo suite [36], using Bowtie version 2.1.0, Tophat v2.0.9 and Cufflinks v2.1.1 with genome assembly GRCh37, finding predominantly Ig gene segments differentially expressed. The final analysis, shown in the figure, employed alignment to the GRCh38 genome build by STAR 2.4.0g1 [37] and gencode v21 exon junctions, giving 13–23 million mapped reads per sample. We then calculated RPKM expression values using rpkmforgenes [38], version 13 Mar 2015 with options -midread -readcount -rmnameoverlap. P-values were calculated using DESeq2 [39], using all genes except microRNA and snoRNA genes, comparing 4 FCRL4+ against 4 FCRL4- samples. We used a 5% Benjamini-Hochberg false discovery rate cutoff. For Ig gene segment expression, we used gencode v22 annotation and selected features with gene_type IG_V_gene, IG_J_gene, IG_C_gene or IG_D_gene, and ran rpkmforgenes with option -midread.

### 2.8 Other statistical analyses

Specific statistical tests used are indicated in the figure legends. Differences were considered to be statistically significant at values of p < 0.05. All statistical analyses were performed by Prism software version 5.0 (GraphPad Software).

## 3 Results

### 3.1 FcRL4+ B cells immunoglobulin genes are enriched for the IgA isotype

**Fig. 1:**
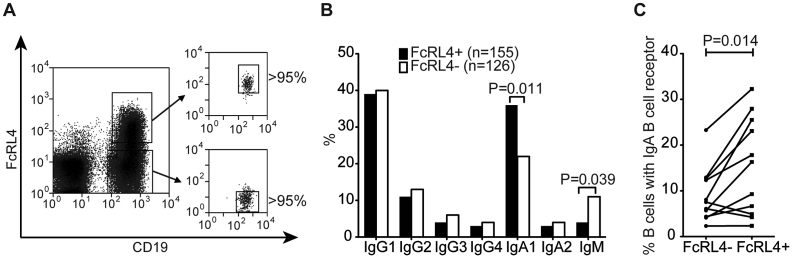
**FcRL4+ B cells are enriched for the IgA Ig subclass**. **(A)** Individual FcRL4+ and FcRL4- CD19+ B cells were sorted for subsequent analysis of their immunoglobulin genes. **(B)** PCR products of IgH chains were sequenced with reverse primers specific for the constant regions of all human Ig classes and the distribution of Ig subclasses in single sorted FcRL4+ and FcRL4- B cells was determined using IMGT database. P values were calculated by χ2 test and corrected for multiple comparisons by the method of false discovery rates. **(C)** Synovial fluid mononuclear cells from RA patients were briefly washed with an acidic buffer (pH 3) to remove externally receptor-bound antibodies and stained for CD19, FcRL4 and IgA. P values were determined by Wilcoxon rank sum test.

Single FcRL4+ and FcRL4- CD19+ B cells were isolated from synovial fluid (n = 4) and synovial tissue (n = 2) from ACPA positive patients with RA (Fig. 1A). Analysis of the Ig isotypes at the gene expression level demonstrated that overall the majority of both FcRL4+ and FcRL4- B cells express IgG (Fig. 1B and supplementary table 2) [27]. However, a significantly higher proportion of FcRL4+ cells expressed IgA1 compared to FcRL4- B cells (*P* = 0.011). Conversely, IgM was expressed in a lower proportion of FcRL4+ B cells compared to FcRL4- B cells (*P* = 0.039). This enrichment of cells expressing IgA B cell receptors in the FcRL4+ subset was also observed at the protein level, by flow cytometry (Fig. 1C).

### 3.2 Synovial FcRL4+ B cells are clonally diverse

**Fig. 2:**
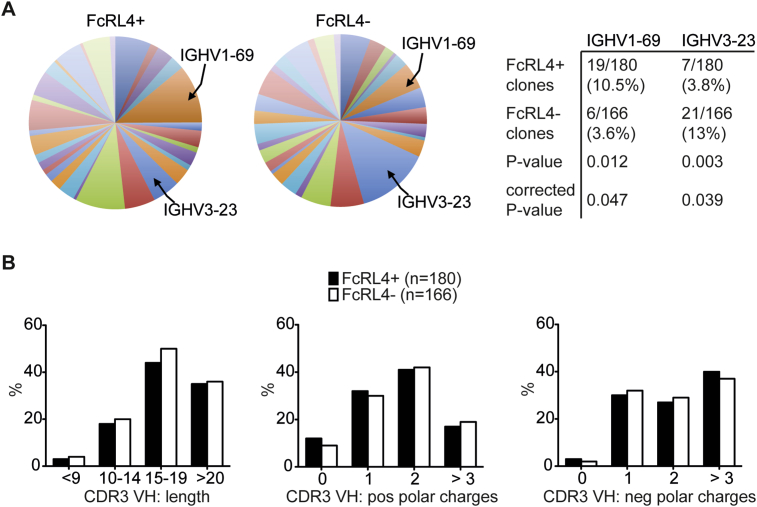
**Investigation of immunoglobulin characteristics of FcRL4+ and FcRL4- B cells**. **(A)**. Ig heavy chain variable (VH) genes of individually sorted FcRL4+ and FcRL4- B cells were sequenced and analyzed by IgBLAST and IMGT/V-QUEST for gene segment repertoire usage. **(B)** Characteristics of IgH complementarity determining region 3 (CDR3) amino acid composition: average length of CDR3s in amino acids, and the mean number of positively and negatively charged amino acids in the CDR3 regions. P values were calculated by χ2 test and corrected for multiple comparisons by the method of false discovery rates.

From the total number of the individually sorted B cells, Ig variable regions of heavy (VH) and light (VL) chain transcripts were successfully amplified by RT-PCR from 346 B cells (180 from FcRL4+ cells and 166 from FcRL4- B cells; supplementary data paper table 2) [27]. Analysis of the variable regions showed that the Ig repertoire was highly diverse in both FcRL4+ and FcRL4- B cells with the majority of the functional Ig genes being represented in the two B cell subsets (Fig. 2A and supplementary data paper tables) [27]. Nevertheless, *VH1-69* was over-represented in the FcRL4+ B cell population (*P* = 0.047), used by 10.5% of FcRL4+ cells compared to 3.6% of FcRL4- B cells. Conversely, *VH3-23* was under-represented in the FcRL4+ B cells compared to FcRL4- B cells (*P* = 0.039 after correction for multiple comparison).

Further analysis of the heavy chain sequences showed that CDR3 features did not significantly differ between FcRL4- and FcRL4+ B cells in terms of amino acid length or frequency of charged residues (Fig. 2B).

### 3.3 Both FcRL4- and FcRL4+ B cells are generated under antigen-mediated selection

**Fig. 3:**
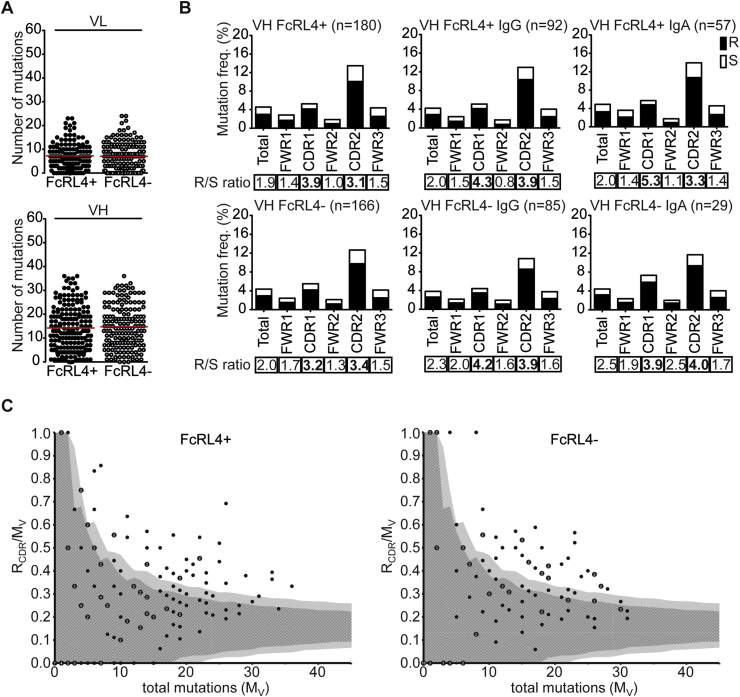
**Mutational analysis in FcRL4+ and FcRL4- B cells**. **(A)** Comparison of the absolute numbers of somatic mutations in individual heavy chain variable (VH) and light chain variable (VL) genes of the antibodies generated from FcRL4+ and FcRL4- B cells. **(B)** Frequencies of replacement (R) and silent (S) mutations in the complementarity determining regions (CDRs) and framework regions (FWRs) of VH regions of FcRL4+ and FcRL4- B cells. The R/S ratios are indicated below the respective graphs. **(C)** Immunoglobulin Analysis Tool (IgAT) analysis to calculate the probability of antigen-driven selection based on somatic mutation analysis. The ratio of replacement mutations in CDR1 and CDR2 (RCDR) to the total number of mutations in V region (MV) was plotted against MV for the FcRL4+ and FcRL4- B cells. The dark and the light grey area indicate the 90% and 95% confidence limits for the probability of random mutations, respectively. A data point outside these areas represents a sequence that was identified as having been selected in an antigen driven process.

Antigen-specific B cell receptor (BCR) genes undergo affinity maturation, typically in the germinal centers, whereby high-affinity B cells accumulate SHMs in their variable region genes [40]. In this study, analysis of SHMs in individual Ig VH and VL genes showed that FcRL4+ and FcRL4- B cells had accumulated equivalent levels of SHMs (Fig. 3A and supplementary data paper table 2) [27].

Based on the frequencies of productive mutations (i.e. the replacement to silent (R/S) mutation ratios) being higher for CDRs compared to FWRs (Fig. 3B) the notion of antigen-mediated selection in germinal centers could be substantiated in both synovial FcRL4+ and FcRL4- B cells.

Additionally, sequences were tested for antigen driven selection using the IgAT Analysis Tool [32] to calculate the probability of antigen-driven selection based on somatic mutation analysis. A similar proportion of sequences from the FcRL4+ and FcRL4- B cell subsets (24.8% and 29.7%, respectively) displayed evidence of antigen-driven selection (Fig. 3C). Taken together, molecular analysis of the Ig genes of both synovial FcRL4+ and FcRL4- B cells suggests that cells in both populations have been differentiated in response to antigen-mediated selection and undergone SHMs and affinity maturation in the context of T cell-dependent immune reactions.

### 3.4 Synovial FcRL4+ B cells contribute to the immune response directed against citrullinated autoantigens

**Fig. 4:**
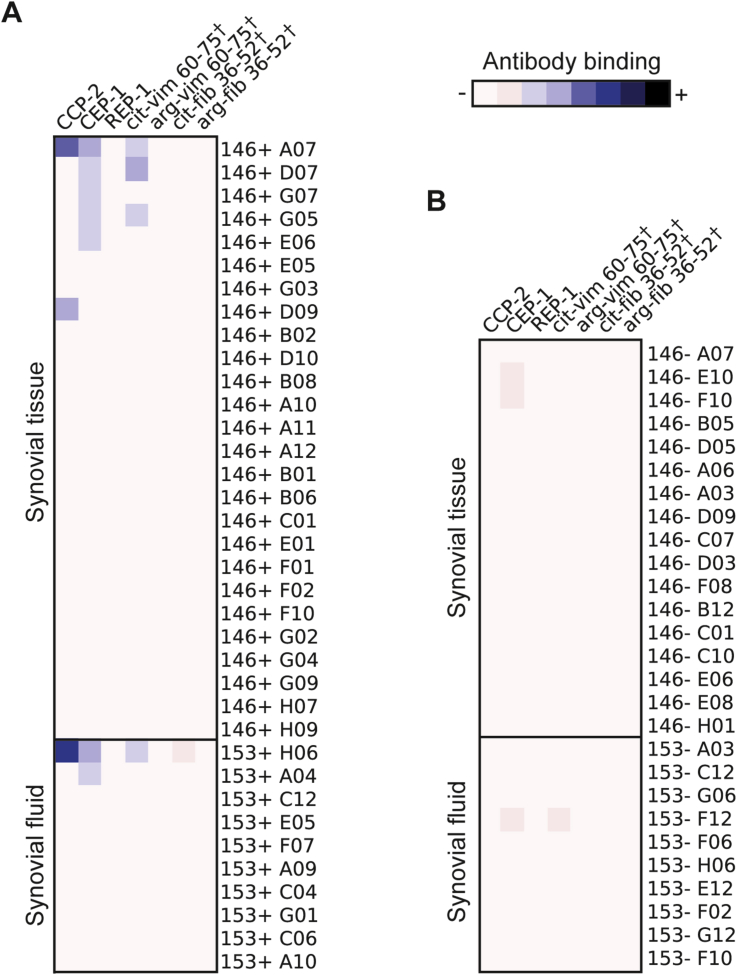
**Heatmaps showing the antigen specificity of recombinant monoclonal antibodies derived from FcRL4+ (A) and FcRL4- (B) B cells**. Monoclonal antibodies were produced by transient transfection in high-density suspension cultures of Expi293F™ cells using PEI-Max. Reactivity with citrullinated peptides was first assessed by use of the commercial cyclic citrullinated peptide (CCP2) test. Reactivity to defined B cell epitopes was then assessed by peptide ELISA for three defined citrullinated peptides and their matching arginine unmodified controls. These were, citrullinated α-enolase peptide 1 (CEP-1), citrullinated vimentin (cit-vim 60–75) and fibrinogen (cit-fib 36–52). The corresponding control peptides were REP-1, arg**-**vim 60–75 and arg-fib 36–52 respectively. The FcRL4+ subset is enriched for B cells specific for citrullinated autoantigens (*P* = 0.018). †, Peptides are designated by their abbreviated protein names (vim for vimentin) followed by the positions of the first and last amino acid. cit, citrulline-containing peptides; arg, native arginine-containing peptides. P value was calculated by Fisher's exact test.

We have previously demonstrated an enrichment of B cells with specificity towards candidate citrullinated autoantigens in the SF of ACPA+ RA patients [4]. Here, the hypothesis that joint-derived B cells with reactivity towards citrullinated antigens would be enriched in the FcRL4+ subset was addressed. For this purpose, the IgH and matching IgL chains from both FcRL4+ and FcRL4- B cells sorted from ACPA+ patients were cloned and transiently expressed, generating recombinant monoclonal antibodies from 36 FcRL4+ B cells and 27 FcRL4- B cells (Fig. 4 and supplementary table 2) [27]. The recombinant monoclonal antibodies were tested for reactivity towards native and citrullinated peptides derived from α-enolase, fibrinogen and vimentin. Eight antibodies were identified as specific for citrullinated antigens and, interestingly, all of these were derived from cells belonging to the FcRL4+ subset (*P* = 0.018) (Fig. 4A and supplementary table 2) [27].

A summary of the characteristics of the antibodies binding citrullinated proteins is also shown in a supplementary table in the supplementary data-only manuscript [27]. (Fig. 4 and supplementary table 2).

Overall, the investigation of the specificity of synovial FcRL4+ B cells for candidate autoantigens showed that these B cells are a component of the autoimmune response to several well-characterized RA-associated, citrullinated autoantigens.

### 3.5 FcRL4+ and FcRL4- synovial B cells have distinct gene expression profiles

**Fig. 5:**
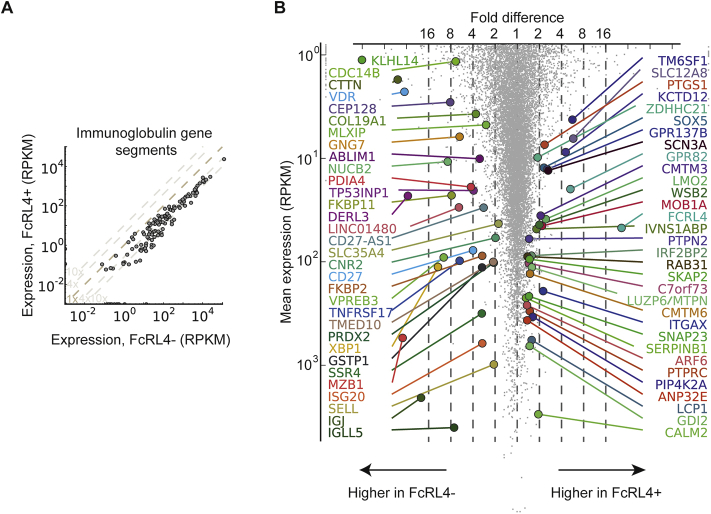
**Synovial fluid FcRL4+ and FcRL4- B cells differ in their gene expression profiles. FcRL4+ and FcRL4- B cells were sorted from RA patients' synovial fluid**. Their gene expression profile was determined by RNAseq. Scatter plots show the expression of individual Ig segments **(A)** and further differentially expressed genes at the genome-wide level (<5% FDR, DESeq2) after Benjamini-Hochberg correction **(B)** for the two groups of B cells. The RNA-seq data has been deposited at Gene Expression Omnibus with accession number GSE94897.

mRNA from sorted FcRL4+ and FcRL4- B cells from SF of ACPA+ RA patients was analyzed by RNA-seq. Data shown in Fig. 5A and B represent the comparison of gene expression between FcRL4+ and FcRL4- B cells. Initial analysis showed that a large proportion of the significant differences in gene expression were found in the immunoglobulin heavy and light chain genes. For clarity, therefore, the immunoglobulin genes are shown in a separate plot (Fig. 5A) with FcRL4+ B cells expressing consistently lower levels of Ig genes, suggesting that these cells are not enriched in cells differentiating towards antibody secreting cells. Fig. 5B shows a scatterplot of the remaining expressed genes, with the genes significantly different after application of the Benjamini-Hochberg correction for multiple comparisons on a genome wide level labeled in color.

**Fig. 6:**
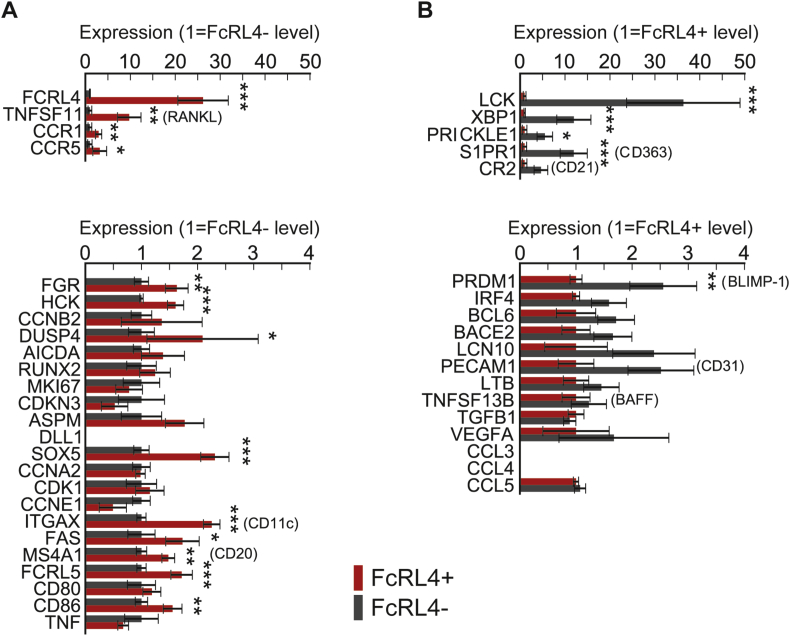
Expression levels of genes previously described as over- or under-expressed in synovial fluid and tonsillar FcRL4+ B cells. (<5% FDR, DESeq2) A) overexpressed in FcRL4+ B) underexpressed in FcRL4+ B cells.

Expression levels of genes previously described as over or under-expressed in SF and tonsillar FcRL4+ B cells were analyzed without the above stringent correction for multiple comparisons, and shown in Fig. 6, respectively, showing very good agreement with the existing data, also confirming the overexpression of RANKL in RA FcRL4+ B cells we and others have previously reported [14], [23]. A number of further genes involved in immune function were found to be upregulated in FcRL4+ B cells here consistent with findings from previous work by us and others. These include CCR1 and CCR5, (receptors for pro inflammatory chemokines which may explain the localization of the FcRL4+ B cells at sites of inflammation) and FcRL5 (which is closely related to FcRL4 and has been suggested to have IgG binding activity) [25]. CD11c is very highly expressed in FcRL4+ B cells and this may affect adhesion and complement binding of FcRL4+ B cells.

Overall, FcRL4+ B cells showed a depletion of plasma cell associated genes when compared with the FcRL4- fraction. Comparative under-expression of transcription factors such as BLIMP-1 and XBP1 in FcRL4+ B cells again supports the view that these cells are not in process of differentiating towards plasma cells, even though they were enriched for the characteristic specificities of RA autoantibodies.

## 4 Discussion

Synovial FcRL4+ B cells are an activated B cell population expressing high levels of RANKL, a key cytokine driving bone erosion and development of lymphoid structures [15]. Hence, FcRL4+ B cells represent a potentially pathogenic B cell subset capable of contributing to the immunopathology of RA.

FcRL4+ B cells were first identified in tonsils, where they accumulate in the vicinity of the epithelium [22], [41]. Furthermore, a small population of these cells was found in the spleen and in reactive lymph nodes [22]. There is little understanding of their function in MALT, but it has become clear that while they are highly proliferative, they show no expression of transcription factors associated with B cell differentiation towards plasma cells [41]. FcRL4 can act as a low affinity IgA receptor; this may be a feature of the role of FcRL4+ B cells in the MALT and potentially also in the joints [25].

This study represents the first investigation of the role of FcRL4+ B cells in the autoimmune response operating in the rheumatoid joints. Analysis of Ig gene sequences of synovial FcRL4+ B cells showed a high level of somatic hypermutation, supporting the view that these cells are post-germinal center memory B cells that have been selected in an antigen and T cell dependent context. Isnardi et al. [42] described an autoreactive B cell population in the peripheral blood of patients with RA, which shares a low level of CD21 expression with the FcRL4+ B cell subset. However, the cells they described were naïve B cells with germline immunoglobulin variable gene segment arrangement. There was no evidence for over-expression of either FcRL4 or RANKL [42] and these cells are clearly distinct from the memory B cells investigated in this project. Furthermore, a population of CD21 low/CD11c high B cells has been observed in peripheral blood of patients with a number of chronic diseases and in advanced age (reviewed by Thorarinsdottir et al. [43]). FcRL4+ cells were not detected in the majority of peripheral blood samples from RA patients, [14]. However, since CD21 low/CD11c high memory B cells share several surface markers with the cells we have investigated, it is possible that they are the precursors of the FcRL4+ B cells we observed in RA joints.

The repertoire of Ig gene segments forming the variable region of B cell receptors was highly diverse in both FcRL4+ and FcRL4- B cells (Fig. 1B). However, there was a statistically significant enrichment of usage of VH1-69 in the FcRL4+ B cell subset. VH1-69 is a gene segment that has previously been associated with autoimmune diseases and is often a component of the variable region of the gene coding for rheumatoid factor [44]. In hepatitis C infected patients with cryoglobulinaemia a potentially related, IgM expressing B cells population has been shown to use VH1-69 [45]. Conversely, VH3-23 was significantly over-represented in the FcRL4- B cells, this may be due to an overall high frequency of B cells using the VH3-23 segment in the synovial tissue as described by Kim et al. [46] and in peripheral blood of healthy controls [47]. Furthermore, it is of interest that autoantigen specific B cells using VH3-23 have been described in healthy controls [48], [49].

In the current study, analysis of Ig isotype usage showed that while the most common isotype of the FcRL4+ B cells was IgG, there was a significant enrichment of IgA expressing B cells in the FcRL4+ compared to the FcRL4- population (Fig. 1). This association of FcRL4+ B cells with IgA is interesting in the light of current hypotheses of the pathogenesis of RA, in which clinical onset can be preceded by mucosal inflammation in the gums, the gut and the lungs [50], [51], [52]. Furthermore, the presence of IgA RF and/or IgA ACPAs is predictive of aggressive disease in RA patients [53], [54]. Further work is needed to investigate whether the IgA immune response in the mucosa generates IgA+ FcRL4+ B cells, which later migrate to the synovium, and contribute to local disease progression.

A key aspect of this study was to determine whether the FcRL4+ B cells are a component of the autoimmune process driving RA. With recent technological developments we were able to generate recombinant monoclonal antibodies from variable regions of Ig heavy and light chain genes of single B cells [29], [30]. This approach was recently used to show the presence of citrulline-reactive B cells in the synovial fluid of ACPA+, but not in ACPA- patients [4], [55], [56]. Our data suggest that FcRL4+ B cells in the synovium are a component of the citrulline-specific autoimmune response in RA.

Global investigation of the gene expression profile of these cells showed that FcRL4+ B cells have a distinct transcriptional profile lacking key regulators of differentiation towards plasma cells (Fig. 5B and supplementary Fig. 2) [27]. Future research should address whether the FcRL4 expressing cells are in a transient stage of differentiation and what their eventual fate is. It is possible that under appropriate stimulatory conditions they will eventually develop into plasma cells. Indeed, Ehrhardt et al. [41] have shown that, if stimulated by IL-2 and IL-10, FcRL4+ B cells from tonsils can be induced to produce antibodies.

Beyond antibody production, several functions of B cells have been described, in particular the production of cytokines including RANKL [13], [23] as well as antigen presentation. Our previous work has demonstrated that synovial FcRL4+ B cells express higher levels of the classical costimulatory molecules, CD80 and CD86, than FcRL4- B cells [14], [41] suggesting that they may function as antigen presenting cells. Given that the citrulline autoreactive B cell receptors were overrepresented in the FcRL4+ B cell subset, these B cells may cross-talk with local T cells and sustain an autoimmune loop within the affected joint. Further work is needed to assess this potential role. Furthermore, in addition to their role in the autoimmune response directed against citrullinated proteins, their high level of RANKL expression suggest that FcRL4+ B cells are a pathogenic B cell subset with a key role in joint destruction and disability in RA.

## 5 Conclusions

B cells identified by their expression of IgA receptor FcRL4 accumulate both in the mucosa associated lymphatic tissue and in the joints of RA patients. We have shown here that FcRL4+ B cells isolated from the joints of RA patients display a significantly increased usage of the IgA isotype. This observation points towards a potential role in the link between mucosal and joint autoimmunity, which requires further investigation.

Importantly, FcRL4+ B cells isolated from the joints of patients with RA are a component of the autoimmune response to citrullinated autoantigens and their RANKL expression suggests that they may contribute to joint damage through cytokine production.

## Competing financial interests

The University of Birmingham has filed a patent covering data from a past publication on FcRL4+ B cells but not on the data shown in this manuscript.
